# Solitary breast metastasis from myxoid liposarcoma

**DOI:** 10.1186/1471-2407-14-482

**Published:** 2014-07-04

**Authors:** Masahiro Yokouchi, Satoshi Nagano, Yuko Kijima, Takako Yoshioka, Akihide Tanimoto, Shoji Natsugoe, Setsuro Komiya

**Affiliations:** 1 Department of Orthopaedic Surgery, Graduate School of Medical and Dental Sciences, Kagoshima University, 8-35-1 Sakuragaoka, Kagoshima 890-8520, Japan; 2 Department of Digestive Surgery, Breast and Thyroid Surgery, Graduate School of Medicine, Kagoshima University, Kagoshima, Japan; 3 Department of Molecular and Cellular Pathology, Graduate School of Medical and Dental Sciences, Kagoshima University, Kagoshima, Japan

**Keywords:** Solitary metastasis, Breast, Resection, Liposarcoma

## Abstract

**Background:**

Metastasis to the breast from nonmammary malignancies is rare, and mostly involves patients in a pre-terminal condition with systemic metastases outside the breast. Lymphoma and leukemia, melanoma, and lung carcinoma are the most common primary malignancies to cause breast metastasis; metastasis of soft tissue sarcoma to the breast is very rare. Here, we report a case of primary lower-extremity myxoid liposarcoma with the development of a solitary metastasis to the breast. To the best of our knowledge, no isolated case reports of solitary breast metastasis by myxoid liposarcoma have been previously reported in the English-language literature.

**Case presentation:**

The patient, a 66-year-old woman, had been previously diagnosed with myxoid liposarcoma of the right thigh. At 21 months after complete surgical resection of the primary tumor with negative margins, a palpable tumor was identified in the patient’s left breast. Needle biopsy revealed the presence of metastatic liposarcoma; positron emission tomography/computed tomography examination confirmed the metastasis as solitary, and no local recurrence of the primary tumor was identified. The patient underwent lumpectomy with negative margins and did not provide consent for adjuvant chemotherapy. As with the biopsy specimen and the total cleavage specimen, myxoid liposarcoma with metastasis to the breast was diagnosed. No recurrence or new metastases were observed five years after resection of the metastatic breast lesion.

**Conclusions:**

We have presented an extremely rare case of a solitary metastatic breast tumor arising from myxoid liposarcoma of the lower limbs. There is no standard treatment for the management of solitary breast metastasis from myxoid liposarcoma. Therefore, treatment should be guided by consideration of an individual patient’s overall condition.

## Background

Breast metastases from nonmammary malignancies are uncommon. Metastases of nonmammary malignancies to the breast represents less than 2% of all breast tumors [1-3]. According to a large recent survey of breast metastases, the most common primary malignancies to cause breast metastasis are lymphoma and leukemia, melanoma, and lung carcinoma; breast metastasis of soft tissue sarcoma is relatively rare [3-5]. Breast metastasis usually indicates disseminated metastatic disease and carries a poor prognosis [2]. Typically, most patients are in a pre-terminal condition with systemic metastases outside the breast [2,3]. Here, we report an extremely rare case of primary lower-extremity myxoid liposarcoma with development of a solitary metastasis to the breast. Five years following resection of the metastatic breast lesion, the patient is without evidence of disease and has a good quality of life.

In our review of the English scientific literature pertaining to solitary breast metastasis arising from myxoid liposarcoma, only two other cases have been reported to date [6,7], but these cases provided either no clinical information [6] or insufficient clinical information [7]. This is the first isolated case report of solitary breast metastasis from myxoid liposarcoma demonstrating clinical findings, clinical management and prognosis. This case report illustrates a pathological rarity and incorporates a brief review of the literature relevant to surgeons.

## Case presentation

**Figure 1:**
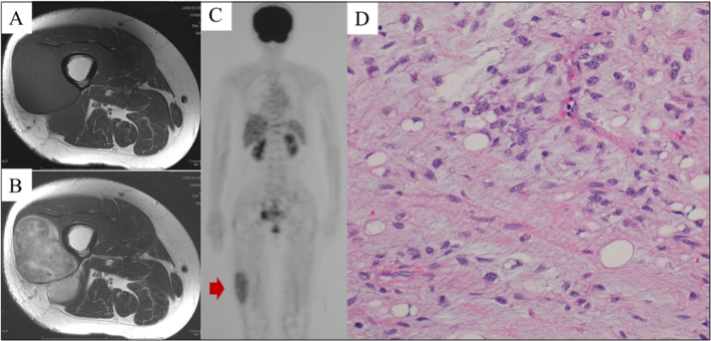
**Radiological images and histological findings of the primary tumor.** Axial **(A)** T1-weighted and **(B)** T2-weighted MRI showed a heterogeneous, ill-defined mass measuring 53 × 48 × 120 mm in the right thigh. **(C)** Maximum intensity projection PET imaging demonstrated slight, heterogeneous ^18^ F-fluorodeoxyglucose uptake in the tumor (SUV = 3.5) (red arrow). Accumulation suggestive of metastasis was not found on PET/CT. **(D)** Microscopic examination of the resected specimen revealed the typical appearance of a myxoid liposarcoma with a large number of mature lipoblasts. No round-cell components were found.

**Figure 2:**
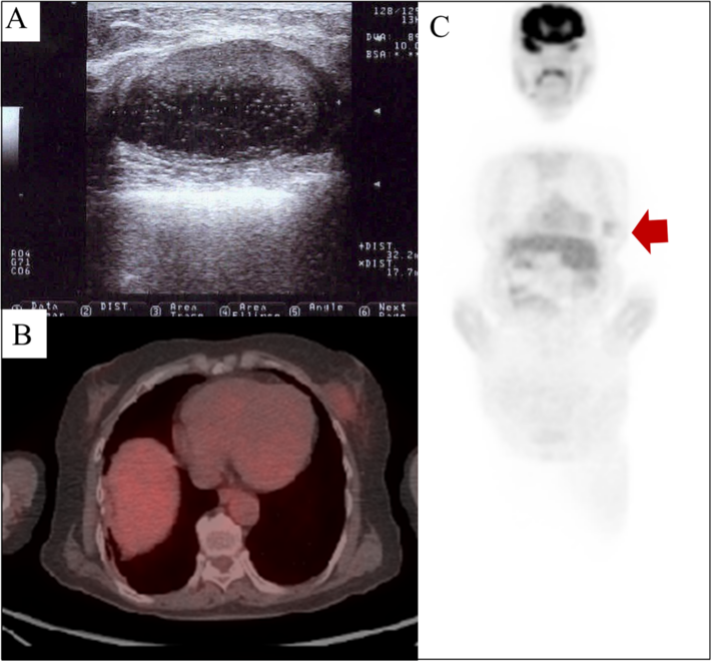
**Radiological images of metastatic breast tumor. (A)** Ultrasonography demonstrated a 3.2 × 2.8 cm irregular, hypoechoic, heterogeneous mass in the breast. Macrocalcifications were not observed. **(B)** Axial PET/CT fusion images revealed the accumulation to be comparatively high (SUV of 2.1) in the tumor within the left breast. **(C)** With the exception of the breast, accumulation suggestive of metastasis was not found on PET/CT.

**Figure 3:**
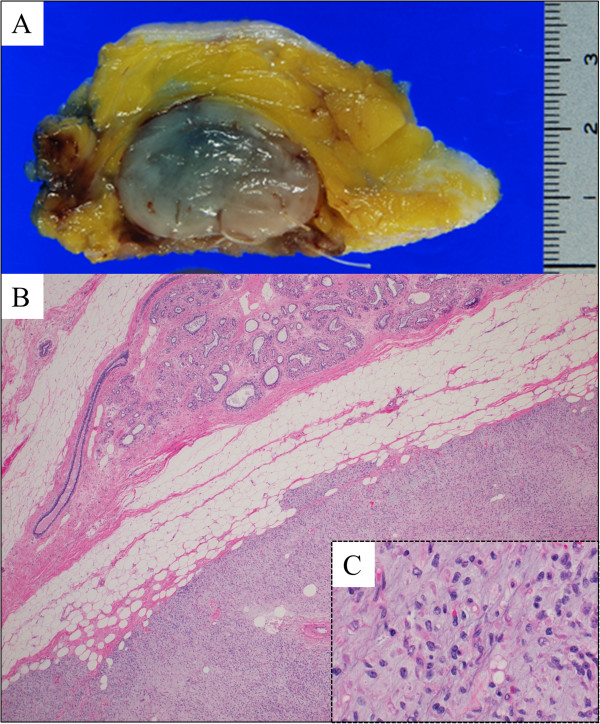
**Macroscopic and histological findings of the excised specimen. (A)** Gross appearance of the resected tissue: a relatively well-circumscribed, multinodular, gelatinous tumor without bleeding or necrosis. The tumor was located in the breast tissue but was close to the pectoralis major muscle. The surface of the pectoralis major muscle was partially resected with the tumor. **(B)** Microscopic examination of the resected specimen revealed the presence of mammary gland tissue surrounding the tumor. **(C)** Lipoblasts were also seen in the resected breast tumor. Histological features of the breast lesion were found to be identical to those of the primary lesion of the extremity. The Estrogen Receptor and Progesterone Receptor status of the histological material was negative.

A 66-year-old woman complained of a progressively enlarging, painful mass over the lateral part of her right thigh for the past 4 months. She had no medical history of note. Magnetic resonance imaging (MRI) revealed a large, well-defined mass located in the region from the anterior to the posterior compartment of the right thigh (Figure 1A, B). Positron emission tomography/computed tomography (PET/CT) revealed significant accumulation of the tracer (standardized uptake value (SUV) of 3.5) in the lesion (Figure 1C). With the exception of the right thigh, no significant accumulation was observed on PET/CT. A biopsy of the mass suggested the presence of a myxoid liposarcoma. After complete surgical resection of the tumor with negative margins, histological examination confirmed the preoperative diagnosis of myxoid liposarcoma (less than 5% round cell component [8,9]) (T2bN0M0G2 : stage IIB) (Figure 1D). Chemotherapy was not administered.At 21 months after surgical resection, the patient consulted a local hospital after noticing a mass in her left breast. She underwent a core needle biopsy. The pathological diagnosis was metastatic liposarcoma, and the patient was subsequently referred to our hospital. On physical examination, a well-circumscribed, mobile, 4 × 3 cm mass was palpable at the 5 o’clock position, close to the areolar border. There was no nipple discharge. Ultrasonography demonstrated a 3.2 × 2.8 cm irregular, hypoechoic, heterogeneous mass in the breast. Macrocalcifications were not observed (Figure 2A). Axial PET/CT fusion images revealed the accumulation to be comparatively high (SUV of 2.1) in the tumor within the left breast (Figure 2B). With the exception of the breast, accumulation suggestive of metastasis was not found on PET/CT (Figure 2C). The patient underwent partial mastectomy with negative margins (1 cm from the edge of the tumor) and did not provide consent for adjuvant chemotherapy. Macroscopically, the resected tissue was a relatively well-circumscribed, multinodular, gelatinous tumor (Figure 3A); as with the biopsy specimen and the hematoxylin and eosin stained total cleavage specimen, metastatic liposarcoma was diagnosed (Figure 3B, C).

The patient’s postoperative course was uneventful. Since most sarcomas recur in the lungs, the patient has been carefully observed with repeated total body CT imaging. No recurrence or new metastases in the lungs or elsewhere were observed in the 5 years after resection of the metastatic lesion in the breast.

## Discussion

Although the breast is the most common site of primary cancer for women, metastatic lesions in the breast are uncommon. Most metastatic breast lesions are metastases from the contralateral breast, but rare metastases from primary malignancies outside the breast can occur [1-4]. The rarity of this occurrence is suggested to be due to the characteristics of breast tissue, as it contains large areas of fibrous tissue with a relatively poor blood supply [2]. This is particularly the case in older women, whose breasts have more fibrous tissue and a poorer blood supply compared with younger women [10]. This anatomical difference also explains why the majority of breast metastases occur in woman younger than 50 years of age [1], while primary malignancies are more common in women older than 50 years of age.

According to the literature, breast metastases occur more frequently in malignant melanomas, sarcomas, lung cancers, ovarian tumors, renal carcinomas, and thyroid tumors [3,11,12]. Previous reports have suggested that breast metastases from different primary tumors have distinct radiological patterns. For instance, the largest lesions occurred in rhabdomyosarcomas, followed by hepatocellular carcinomas and squamous cell carcinomas of the head and neck region. The smallest lesions occurred in thyroid gland carcinomas [13]. Most breast metastases showed circumscribed margins, while metastases from rhabdomyosarcomas were microlobulated [13]. On ultrasound, breast lesions in lung cancers were usually inhomogeneously hypoechoic with circumscribed margins and demonstrated posterior shadowing in almost 50% of the cases. Breast metastases from ovarian carcinomas have typically microlobulated margins and posterior enhancement [13].

Soft tissue sarcoma is a rare tumor that accounts for less than 1% of all malignant neoplasms in humans. Myxoid liposarcoma is the second most common subtype of liposarcoma and represents approximately 10% of all adult soft tissue sarcoma [8,9]. Approximately one-third of patients with myxoid liposarcoma develop distant metastasis, which carries a poor prognosis, and it is well documented that myxoid liposarcoma has an unusual metastatic pattern. Myxoid liposarcoma tends to metastasize to extrapulmonary sites, such as soft tissue of the opposite side and bone, without causing pulmonary metastases; in contrast, most of the histological types of soft tissue sarcoma metastasize hematogenously to the lungs [8,9].

Regarding metastasis to the breast, a few cases of breast metastasis from myxoid liposarcoma have been reported in the previous literature [6,7,14,15]. However, even with myxoid liposarcoma, breast metastasis is mostly a result of disseminated systemic metastasis outside the breast, and solitary metastasis to the breast is extremely rare. To the best of our knowledge, no isolated case reports of solitary breast metastasis from myxoid liposarcoma have been previously reported in the English-language literature. In our review of publications written in English, we were only able to identify two cases of solitary metastasis to the breast from myxoid liposarcoma [6,7]. One report briefly mentions the existence of breast metastases from myxoid liposarcoma without any information about the patient [6]. The other report described solitary metastasis to the breast from myxoid liposarcoma of the thigh developing four years after the primary diagnosis [7]. However, the age, gender, radiological and pathological findings, treatment strategy and prognosis of the patients were not described [7]. The characteristics and treatment strategies of solitary breast metastasis from myxoid liposarcoma have not been fully ascertained.

At present, conservative treatment with the systemic administration of anticancer agents in conjunction with radiation therapy is often chosen to treat metastatic lesions, and the necessity of surgical treatment remains controversial. As previously mentioned, breast metastasis usually indicates disseminated metastatic disease and a poor prognosis [2,16]. Yeh et al. reported that, in their series, over 90% of breast metastases were associated with disseminated metastatic disease, with most patients dying within a year of the diagnosis of breast metastasis; the median survival was 4 months in this study [2]. Amichetti et al. also described the development of breast metastasis as a negative prognostic factor, and they concluded that recognition of breast metastasis is useful in avoiding unnecessary radical operative procedures [16].

In general, metastatic tumors usually require treatment according to the origin and type of the tumor. Regarding myxoid liposarcoma, the disease-free interval and the overall survival rate have been shown to be significantly better for patients with extrapulmonary metastases compared with those with pulmonary metastases [6,7]. Spillance et al. analyzed the natural history of soft tissue metastasis from myxoid liposarcoma and concluded that soft tissue metastases should be managed aggressively, most often involving further surgery [17]. The impact of chemotherapy on patients with metastatic myxoid liposarcoma remains controversial. Therefore, in the present case, we decided to perform surgical treatment.

There is no consensus regarding the treatment of solitary breast metastasis from myxoid liposarcoma, so clinical decisions must be formulated on a case-by-case basis. As a result of surgical treatment, our patient is alive with no evidence of disease five years after resection of the metastatic breast lesion. Curative surgical resection appears to have been an effective treatment choice in this case.

Regarding radiotherapy, myxoid liposarcoma is known to be particularly sensitive to radiotherapy compared with other histologic subtypes of soft tissue sarcoma [18]. Therefore, radiotherapy has been widely used in combination with surgery for cases in which adequate surgical resection is not feasible. Because of the rarity of the clinical scenario in our case, it is difficult to compare the validity of the resection that we performed for the metastatic lesion with radiation therapy as a local therapy. More cases are required in order to identify the ideal treatment protocol for solitary breast metastasis from myxoid liposarcoma.

PET/CT is widely used for staging in various malignancies, especially for nodal and distant metastasis staging. In general, sarcomas tend to be 18 F-fluorodeoxyglucose avid and whole-body PET/CT is described as an ideal modality for staging malignant soft tissue sarcomas [9]. However, several recent studies have reported wide-ranging sensitivities and specificities for this method of detection of metastatic soft tissue sarcoma [19]. Although we used PET/CT in the preoperative evaluation of this patient, the utility of PET/CT for the staging of soft tissue sarcoma remains to be defined [19].

Concerning the concept of multicentric liposarcoma [9], debate still persists as to whether multifocal myxoid liposarcoma represents metastatic disease or independently arising, multicentric primary disease. To formulate an optimal treatment plan for patients, it might be important for a clinician to distinguish between metastasis and multicentric primary disease. While in the future it may be possible to make this distinction by molecular analysis of the tumor [20], at present the most plausible explanation for the overwhelming majority of cases of multifocal myxoid liposarcoma is metastatic disease [9].

## Conclusions

We have presented an extremely rare case of a solitary metastatic breast tumor arising from myxoid liposarcoma of the lower limbs. Although treatment experience is limited owing to the rarity of this condition, our case report illustrates that, depending on a patient’s overall condition, complete curative resection might be considered a valid therapeutic option when treating myxoid liposarcoma with breast metastasis.

### Ethical adherence

This study was performed in accordance with the Helsinki Declaration and the written consent by the patient.

### Consent

Written informed consent was obtained from the patient for publication of this case report and any accompanying images. A copy of the written consent is available for review by the Editor of this journal.

## Abbreviations

MRI: Magnetic resonance imaging; CT: Computed tomography; PET/CT: Positron emission tomography/computed tomography; SUV: Standardized uptake value.

## Competing interests

The authors declare that they have no competing interests.

## Authors’ contributions

MY, SaN, YK and ShoN participated in the surgical treatment and follow-up of the patient. MY and SK drafted and finalized the manuscript. TY and AT performed pathological examination and figure preparation. All authors have read and approved the final manuscript.

## Pre-publication history

The pre-publication history for this paper can be accessed here:

http://www.biomedcentral.com/1471-2407/14/482/prepub
